# Prokineticin 2 potentiates acid-sensing ion channel activity in rat dorsal root ganglion neurons

**DOI:** 10.1186/1742-2094-9-108

**Published:** 2012-05-29

**Authors:** Chun-Yu Qiu, Yu-Qiang Liu, Fang Qiu, Jiliang Wu, Qun-Yong Zhou, Wang-Ping Hu

**Affiliations:** 1 Department of Pharmacology, Hubei University of Science and Technology, 88 Xianning Road, Xianning, Hubei, 437100, People's Republic of China; 2 The Laboratory of Cardiovascular, Cerebrovascular, and Metabolic Disorder, Hubei University of Science and Technology, 88 Xianning Road, Xianning, Hubei, 437100, People's Republic of China; 3 Department of Pharmacology, University of California Irvine, Irvine, CA, USA

**Keywords:** Acid-sensing ion channel, Dorsal root ganglion neuron, Neuronal excitability, Pain, Prokineticin 2, Proton-gated current

## Abstract

**Background:**

Prokineticin 2 (PK2) is a secreted protein and causes potent hyperalgesia in vivo, and is therefore considered to be a new pronociceptive mediator. However, the molecular targets responsible for the pronociceptive effects of PK2 are still poorly understood. Here, we have found that PK2 potentiates the activity of acid-sensing ion channels in the primary sensory neurons.

**Methods:**

In the present study, experiments were performed on neurons freshly isolated from rat dorsal root ganglion by using whole-cell patch clamp and voltage-clamp recording techniques.

**Results:**

PK2 dose-dependently enhanced proton-gated currents with an EC_50_ of 0.22 ± 0.06 nM. PK2 shifted the proton concentration-response curve upwards, with a 1.81 ± 0.11 fold increase of the maximal current response. PK2 enhancing effect on proton-gated currents was completely blocked by PK2 receptor antagonist. The potentiation was also abolished by intracellular dialysis of GF109203X, a protein kinase C inhibitor, or FSC-231, a protein interacting with C-kinase 1 inhibitor. Moreover, PK2 enhanced the acid-evoked membrane excitability of rat dorsal root ganglion neurons and caused a significant increase in the amplitude of the depolarization and the number of spikes induced by acid stimuli. Finally, PK2 exacerbated nociceptive responses to the injection of acetic acid in rats.

**Conclusion:**

These results suggest that PK2 increases the activity of acid-sensing ion channels via the PK2 receptor and protein kinase C-dependent signal pathways in rat primary sensory neurons. Our findings support that PK2 is a proalgesic factor and its signaling likely contributes to acidosis-evoked pain by sensitizing acid-sensing ion channels.

## Background

It is well known that tissue acidosis produces pain. For instance, direct application of an acidic solution into the skin induces non-adapting pain [1,2]. Tissue acidosis occurs in a variety of pathological states, including inflammation, ischemia, tissue injury and tumor development [3,4]. The local drop in pH is detected by acid-sensing ion channels (ASICs) on peripheral nociceptors and plays an important role in pathological pain in these conditions [5]. To date, seven subunits of ASICs (1a, 1b1, 1b2, 2a, 2b, 3 and 4) encoded by four genes have been identified [6]. All ASICs except ASIC4 are present in primary sensory neurons [7,8]. ASICs are activated by extracellular protons and contribute to pain caused by tissue acidosis. Increasing evidence suggests that ASICs are involved in inflammatory and neuropathic pain [9-11].

**Figure 1:**
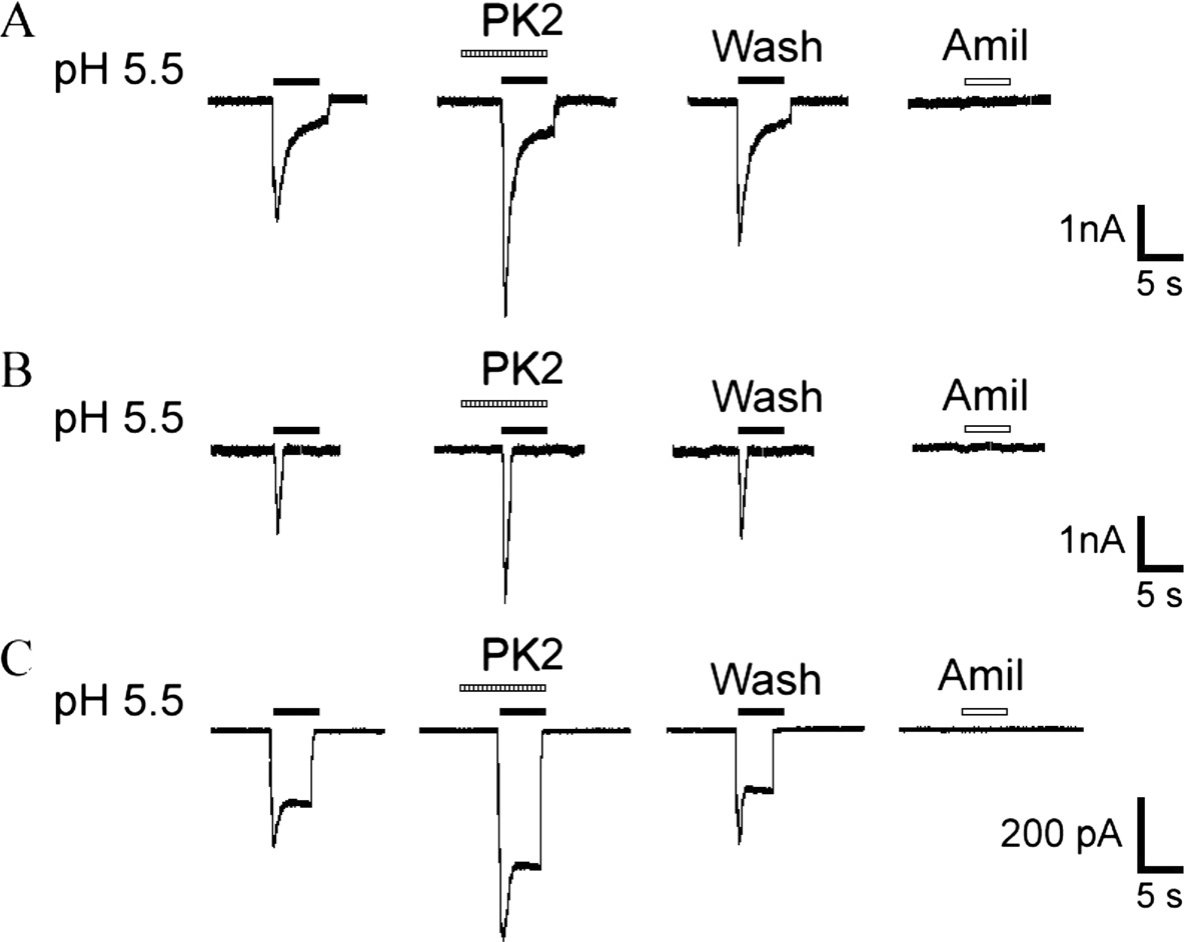
**Potentiation of proton-gated currents by prokineticin 2 in rat dorsal root ganglia neurons.** Three types of proton-induced currents were recorded in DRG neurons. Example traces of a **(A)** slow-inactivating current, **(B)** fast-inactivating current and **(C)** sustained current. All three types of proton-induced currents could be inhibited by 20 μM amiloride, a broad-spectrum ASIC channel blocker. Pre-application of PK2 increased the peak phases of all three types of proton-induced currents. Proton-induced currents were evoked by extracellular application of a pH 5.5 solution for 5 s in the presence of the TRPV1 inhibitor capsazepine (10 μm). The pretreatment time for PK2 (1 nM) was 60 s. ASIC: acid-sensing ion channels; DRG: dorsal ganglia root; PK2: prokineticin 2; TRPV1: transient receptor potential vanilloid receptor-1.

**Figure 2:**
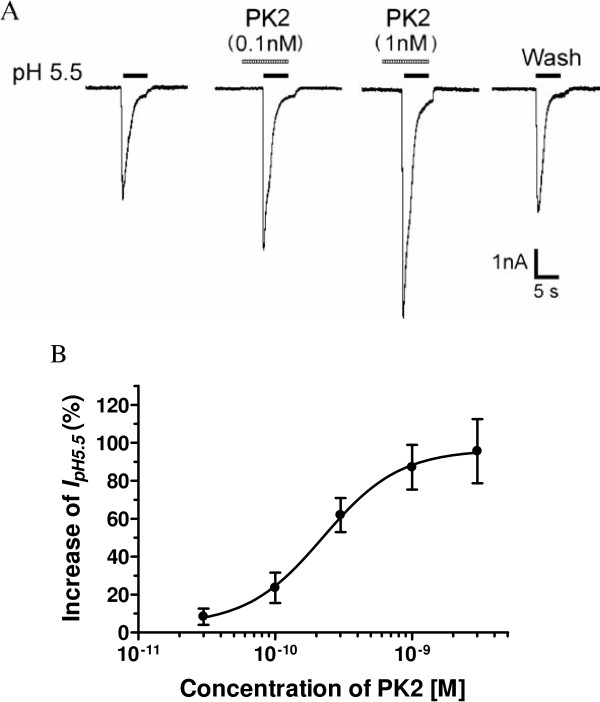
**Concentration-dependent potentiation of proton-gated currents by prokineticin 2. (A)** Sequential current traces illustrate the potentiation of proton-induced currents by different concentration of PK2 on a DRG neuron. Proton-gated currents were elicited by application of pH 5.5 for 5 s durations. The pretreatment time for PK2 was 60 s. **(B)** PK2 concentration-dependently potentiated proton-gated currents (I_pH5.5_). Each point represents the mean ± SEM of 6 to 10 neurons. DRG: dorsal ganglia root; PK2: prokineticin 2.

**Figure 3:**
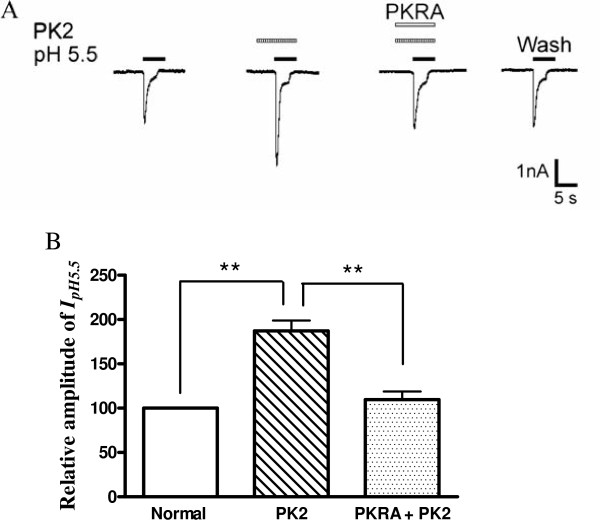
**Blockade of prokineticin 2-induced potentiation of proton-gated currents by prokineticin 2 receptor antagonist.** The current traces in **(A)** and the bar graph in **(B)** show that the potentiation of I_pH5.5_ by PK2 (1 nM) pre-applied alone was abolished by the co-application of PK2 and PKRA (10 nM), a PK2 receptor antagonist. **P <0.01, one-way analysis of variance followed by post hoc Bonferroni’s test, n = 7. PKRA: prokineticin 2 receptor antagonist; PK2: prokineticin 2.

**Figure 4:**
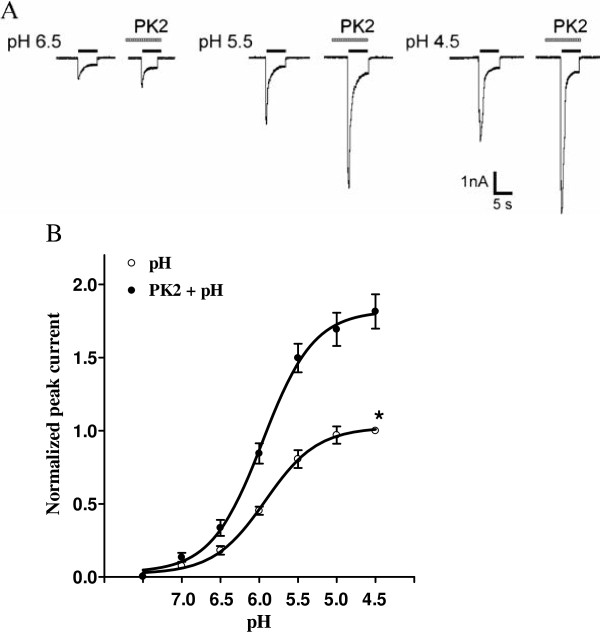
**Concentration-response relationship for protons with or without the pre-application of prokineticin 2. (A)** Sequential currents evoked by different pHs in the absence or presence of PK2. **(B)** The concentration-response curves for protons with or without PK2 (1 nM) pre-application. Each point represents the mean ± SEM of 7 to 11 neurons. All current values were normalized to the current response induced by pH 4.5 applied alone (marked with asterisk). The curves shown are a best fit of the data to the logistic equation *I* = *I*_max_/[1 + (EC_50_/*C*)^n^], where *C* is the concentration of protons, *I* is the normalized current response value, EC_50_ is the proton concentration that produced half the maximal current response to protons, and *n* is the Hill coefficient. The curves for protons without and with PK2 pre-application were drawn according to the equation described above (the Hill coefficients are 1.29 with PK2 pre-application and 1.31 without). The pretreatment of PK2 shifts the concentration-response curve for protons upwards. PK2: prokineticin 2; SEM: standard error of the mean.

**Figure 5:**
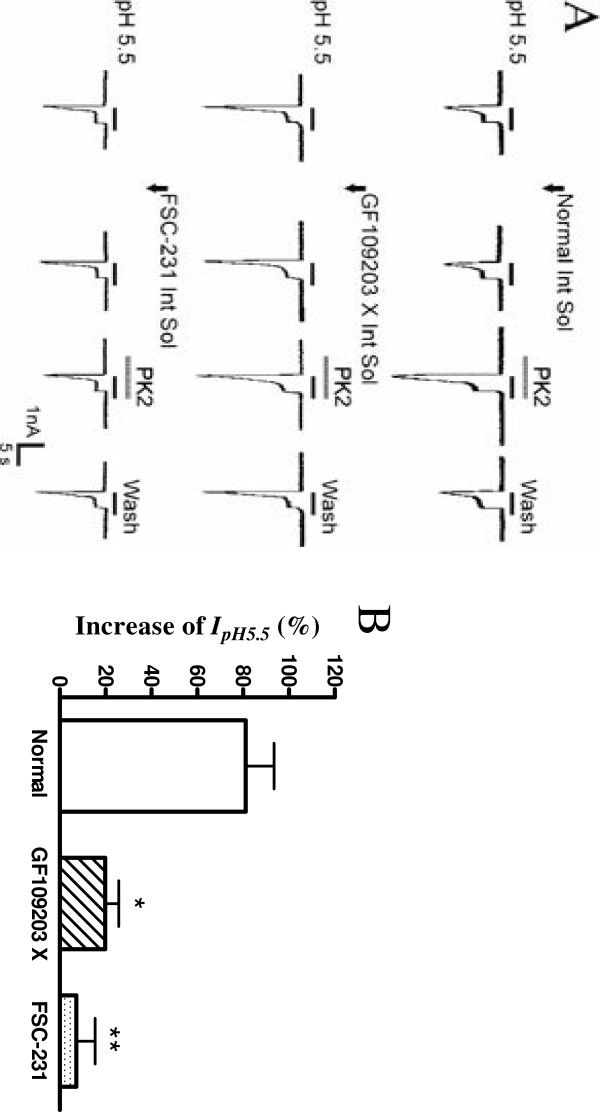
**Intracellular mechanisms underlying the potentiation of proton-gated currents by prokineticin 2 signaling. (A)** The first line of current traces show that PK2 still enhanced proton-gated currents after re-patch with normal internal solution on the same neuron. The second line of current traces show the effect of intracellular dialysis of GF109203X, a PKC inhibitor, on the PK2-induced potentiation of proton-gated currents. The third line of traces were taken from neurons treated with PICK1 inhibitor FSC-231. GF109203X (2 μM) and FSC-231 (50 μM) was included in the recording pipette for intracellular dialysis. Proton-gated currents were elicited by application of pH 5.5 for 5 s durations. PK2 (1 nM) was pre-applied to external solution for 1 min. Arrow represents re-patch clamp and intracellular dialysis. The bar graph in **(B)** shows the percentage increases in the I_pH5.5_ induced by PK2 (10^-9^ M) with recording pipettes filled with the normal internal solution (n = 7), GF109203X (n = 8) or FSC-231 (n = 8) containing internal solution in the second patch experiments. Intracellular dialysis of GF109203X or FSC-231 abolished the enhancing effect of PK2 on I_pH5.5_. *P <0.05, **P <0.01, post hoc Bonferroni’s test, compared with normal internal solution. PICK1: protein interacting with C-kinase 1; PK2: prokineticin 2.

**Figure 6:**
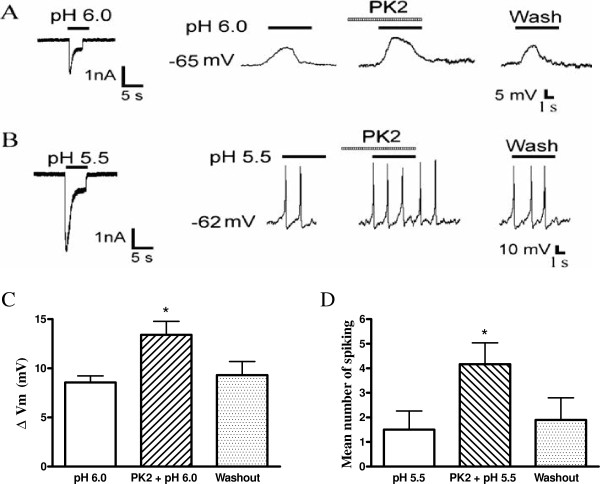
**Effect of prokineticin 2 on proton-evoked membrane excitability of rat dorsal root ganglia neurons. (A)** Original current and membrane potential recordings from the same DRG neuron. Left panel: voltage-clamp recording of current induced by a pH 6.0 acid stimulus in the presence of the TRPV1 inhibitor capsazepine (10 μm). Holding potential was −60 mV. Right panel: current-clamp recording (I = 0 pA) of the depolarization evoked by the pH 6.0 acid stimulus from the same neuron as the left panel in the presence of capsazepine (10 μM) to block TRPV1 and TTX (1 μM) to block Na^+^ channel-mediated action potentials. The pretreatment of PK2 (1 nM) enhanced the acid-induced membrane depolarization. No action potential was triggered by the membrane depolarization in the neuron. **(B)** Original current and spiking recordings from the same DRG neuron. Left panel: a pH 5.5 acid stimulus induced an inward current with voltage-clamp recording. Right panel: the pH 5.5 acid stimulus produced a cell spiking with current-clamp recording in the same neuron in the presence of the TRPV1 inhibitor capsazepine (10 μM). The pretreatment with PK2 (1 nM) increased the acid-induced spiking activity. **(C and D)** Bar graphs show the effect of PK2 on membrane potential depolarization produced by pH 6.0 and the number of spiking produced by pH 5.5. After a 20 min washout of PK2, the acid-evoked depolarization and spiking recovered to control condition. *P <0.05, paired *t*-test, compared with pH alone, n = 6 in each column. DRG: dorsal ganglia root; PK2: prokineticin 2; TRPV1: transient receptor potential vanilloid receptor-1; TTX: tetrodotoxin.

**Figure 7:**
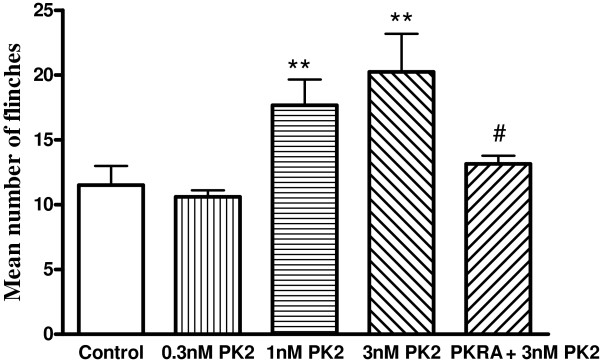
**Effect of prokineticin 2 on nociceptive responses to injection of acetic acid in rats.** Intraplantar injection acetic acid evoked a flinch/shaking response in the presence of the TRPV1 inhibitor capsazepine (100 μM). The pretreatment of PK2 increased flinching behavior induced by acetic acid in dose-dependent manner. The effect of PK2 was blocked by PKRA (10 nM), a PK2 receptor antagonist. Flinching or shaking of the paw was recorded as the number of flinches per observation period (5 min). **P <0.01, post hoc Bonferroni’s test, compared with control; ^#^*P* <0.05, post hoc Bonferroni’s test, compared with PK2 (3nM) column. n = 10 per each column. PKRA: prokineticin 2 receptor antagonist; PK2: prokineticin 2; TRPV1: transient receptor potential vanilloid receptor-1.

Prokineticin 2 (PK2) is a multifunctional secreted regulatory peptide that signals through two G protein-coupled receptors termed prokineticin receptors 1 and 2 (PKR1 and PKR2) [12-14]. Emerging evidence indicates that PK2 is also associated with pain perception. Intraplantar injection of PK2 and Bv8, the frog homolog of mammalian PK2, causes a strong and localized hyperalgesia by reducing the nociceptive thresholds to thermal, mechanical and chemical stimuli [15-17]. PK2 is highly expressed by neutrophils and other inflammatory cells and releases in inflamed tissues, thus it is considered as a new pronociceptive mediator [18].

Genetic studies have confirmed the critical involvement of PK2 signaling in pain perception, particularly via PKR1 activation. Mice lacking either PK2 or PKR1 genes displayed a significant reduction in nociception induced by noxious heat and various chemical stimuli such as capsaicin, protons and formalin [15,19]. Recently, Watson et al. found that increased PK2 expression in gut inflammation likely contributes to visceral pain [20]. It has been shown that PKR1 and PKR2 are present in nociceptive primary sensory neurons [15,17]. Calcium image studies have revealed that the majority of dorsal root ganglia (DRG) neurons are activated by PK2 [15,17]. Thus, PK2 may act directly on sensory neurons. It has been shown that Bv8 sensitizes transient receptor potential vanilloid receptor-1 (TRPV1) and potentiates the inward current activated by capsaicin in DRG neurons [21]. These results demonstrate a positive interaction between the PKRs and the vanilloid receptors. However, blocking TRPV1 pathway reduces but does not abolish Bv8-induced hyperalgesia, indicating that Bv8-induced pain sensitization also involves other signaling pathways [19,22]. In this study, we show that PK2 potentiates the activity of ASICs in the rat DRG sensory neurons.

## Methods

### Isolation of the dorsal root ganglia neurons

The experimental protocol was approved by the animal research ethics committee of Hubei University of Science and Technology. All procedures conformed to international guidelines on the ethical use of animals, and every effort was made to minimize the number of animals used and their suffering. Two- to three-week old Sprague–Dawley male rats were anesthetized with ethyl ether and then decapitated. The DRGs were taken out and transferred immediately into DMEM (Sigma-Aldrich, St. Louis, MO, US) at pH 7.4. After the removal of the surrounding connective tissues, the DRGs were minced with fine spring scissors and the ganglion fragments were placed in a flask containing 5 mL of DMEM in which trypsin (type II-S, Sigma) 0.5 mg/mL, collagenase (type I-A, Sigma) 1.0 mg/mL and DNase (type IV, Sigma) 0.1 mg/mL had been dissolved, and incubated at 35 °C in a shaking water bath for 25 to 30 min. Soybean trypsin inhibitor (type II-S, Sigma) 1.25 mg/mL was then added to stop trypsin digestion. Dissociated neurons were placed into a 35 mm Petri dish and kept for at least another 60 min before electrophysiological recordings. The neurons selected for electrophysiological experiment were 15 to 35 μm in diameter.

### Electrophysiological recordings

Whole-cell patch clamp and voltage-clamp recordings were carried out at room temperature (22 to 25 °C) using a MultiClamp-700B amplifier and Digidata-1440A A/D converter (Axon Instruments, Foster City. CA, USA). Recording pipettes were pulled using a Sutter P-97 puller (Sutter Instruments, Novato, CA, USA). The micropipettes were filled with internal solution containing KCl 140 mM, MgCl_2_ 2.5 mM, HEPES 10 mM, EGTA 11 mM and ATP 5 mM; its pH was adjusted to 7.2 with KOH and osmolarity was adjusted to 310 mOsm/L with sucrose. Cells were bathed in an external solution containing NaCl 150 mM, KCl 5 mM, CaCl_2_ 2.5 mM, MgCl_2_ 2 mM, HEPES 10 mM and d-glucose 10 mM; its osmolarity was adjusted to 330 mOsm/L with sucrose and pH to 7.4. The resistance of the recording pipette was in the range of 3 to 6 MΩ. A small patch of membrane underneath the tip of the pipette was aspirated to form a gigaseal and then negative pressure was applied to rupture it, thus establishing a whole-cell configuration. The adjustment of capacitance compensation and series resistance compensation was done before recording the membrane currents. The membrane voltage was maintained at −60 mV in all voltage-clamp experiments unless otherwise specified. Current-clamp recordings were obtained by switching to current-clamp mode after a stable whole-cell configuration was formed in voltage-clamp mode. Only cells with a stable resting membrane potential (more negative than −−50 mV) were used in the study. Signals were sampled at 10 to 50 kHz and filtered at 2 to 10 kHz, and the data were stored in compatible PC computer for off-online analysis using the pCLAMP 10 acquisition software (Axon Instruments).

### Drug application

Drugs used in the experiments include: HCl, PK2 and PK2 receptor antagonist (PKRA; Zhou laboratory, University of California, Irvine, CA, USA), amiloride (Sigma), capsazepine (RBI, Natick, MA, US) and tetrodotoxin (TTX; Sigma). All drugs were dissolved daily in the external solution just before use and held in a linear array of fused silica tubes (outside diameter/inside diameter = 500 μm/200 μm) connected to a series of independent reservoirs. The application pipette tips were positioned approximately 30 μm away from the recorded neurons. The application of each drug was driven by gravity and controlled by the corresponding valve, and rapid solution exchange could be achieved within about 100 ms by shifting the tubes horizontally with a PC-controlled micromanipulator. Cells were constantly bathed in normal external solution flowing from one tube connected to a larger reservoir between drug applications. In some experiments where GF109203X (RBI) and FSC-231 (Gether Lab, University of Copenhagen, DK-2200 Copenhagen, Denmark) were applied for intracellular dialysis, they were dissolved in the internal solution before use. To functionally characterize ASIC activity, we used capsazepine (10 μM) to block TRPV1 in this study [23].

### Nociceptive behavior induced by acetic acid in rats

Sprague–Dawley male rats (3 to 4 weeks old) were kept with a 12 h light/dark cycle and with ad libitum access to food and water. Animals were placed in a 30 × 30 × 30 cm^3^ Plexiglas chamber and allowed to habituate for at least 30 min before nociceptive behavior experiments. After pretreatment with 10 μL capsazepine (100 μM), a double-blind experiment was carried out. Twenty microliters of acetic acid solution (0.6%) together with 20 μL external solution, amiloride, PK2 and/or PKRA were coded, and the other experimenters subcutaneously administered them into the dorsal face of the hind paw using a 30 gauge needle connected to a 100 μL Hamilton syringe. Nociceptive behavior (that is, number of flinches) was counted over a 5 min period starting immediately after the injection [9,24].

### Data analysis

Data were statistically compared using the Student’s *t*-test or analysis of variance (ANOVA), followed by Bonferroni’s *post hoc* test. Statistical analysis of concentration-response data was performed using nonlinear curve-fitting program ALLFIT. Data are expressed as mean ± standard error of the mean (SEM).

## Results

### Prokineticin 2 enhanced proton-gated currents in rat dorsal root ganglia neurons

All current measurements in this study were done in small and medium diameter (15 to 35 μm) acutely isolated DRG neurons of adult rats. To functionally characterize ASIC currents, we measured proton-gated currents in the presence of the TRPV1 inhibitor, capsazepine (10 μM), in the whole-cell patch-clamp configuration [23]. In the present study, the majority of DRG neurons (68.9%, 91 out of 132) examined were sensitive to extracellular application of a pH 5.5 solution for 5 s with an inward current (I_pH5.5_). Under our experimental conditions, proton-induced currents could be separated into the following three classes on the basis of their amplitudes and inactivation time courses: a slow-inactivating transient inward current followed by a sustained component was present in 59.3% (54 out of 91) of neurons (Figure 1A); a fast-inactivating transient current was present in 12.1% (11 out of 91) of neurons (Figure 1B); and a sustained current with a small transient phase in 28.6% (26 out of 91) of cells (Figure 1 C). The maximum amplitude of the slow- and rapid-inactivating transient current was at least three times greater than that of the sustained current. The transient currents could be clearly divided into two groups on the basis of their inactivation time constants. The slow-inactivating transient current had a mean inactivation time constant of 1,845.1 ± 192.6 ms, whereas the rapid-inactivating current had a shorter inactivation time constant (452.5 ± 68.3 ms, *P* <0.001). We established objective criteria for the distinction of these current types. Time constants of inactivation <800 ms were classified as rapid-inactivating current, while those >800 ms were classified as slow-inactivating current. In the presence of the TRPV1 inhibitor, capsazepine (10 μm), ASICs are the only channels known to mediate transient proton-gated currents, since all observed transient currents could be inhibited by 20 μM amiloride, a broad-spectrum ASIC channel blocker. Therefore, we considered them as ASIC currents.

Application of PK2 (1 nM) 60 s prior to acid solution enhanced I_pH5.5_ in the majority of the neurons sensitive to acid stimuli (72.5%, 66 out of 91; Figure 1). The enhanced effect of PK2 was observed in all three types of proton-evoked currents (Figure 1). The amplitude of peak phase of slow-inactivating current increased to 187.1 ± 11.8% of that before application of PK2 (n = 7, *P* <0.01), the rapid-inactivating current to 181.4 ± 13.9% (n = 7, *P* <0.01) and the sustained current to 179.4 ± 14.6% (n = 7, *P* <0.01). Since PK2 showed similar modification on all three types of currents, we pooled data in further experiments.

We next investigated whether the potentiation of proton-gated currents was dependent on the concentrations of PK2. Figure 2A shows that the amplitudes of I_pH5.5_ increased when the concentrations of PK2 increased from 0.1 nM to 1 nM. Figure 2B shows the dose–response curve for PK2 in the potentiation of proton-gated currents. PK2 caused the maximum effect (95.6 ± 16.8%, n = 7) at a concentration of 3 nM. The half-maximal response (EC_50_) value of the dose–response curve for PK2 was 0.22 ± 0.06 nM (Figure 2B).

### Blockade of prokineticin 2-induced potentiation of proton-gated currents by prokineticin 2 receptor antagonist

To verify whether the potentiation of proton-gated currents by PK2 was mediated by a PK2 receptor, we used a small-molecule PKRA that blocks both PKR1 and PKR2 [25]. The effect of PKRA on potentiation of proton-gated currents by PK2 was examined. The potentiation of I_pH5.5_ by pretreatment with PK2 (1 nM) was almost completely reversed by the addition of 10 nM PKRA (Figure 3, *P* <0.01, one-way ANOVA followed by Bonferroni’s post hoc test, n = 7). Moreover, PKRA itself had no effect on I_pH5.5_ (data not shown).

### Concentration-response relationship for proton-gated currents with and without pretreatment of prokineticin 2

We then investigated whether the potentiation of PK2 was dependent on the concentrations of proton. Figure 4A shows that currents evoked by different pHs were enhanced by PK2. First, pretreatment of PK2 shifted the concentration-response curve to protons upwards, as indicated by a 1.81 ± 0.11 fold increase of the maximal current response to protons when PK2 was pre-applied. However, the slopes or Hill coefficients of those two curves were essentially similar (n = 1.29 in the absence of PK2 versus n = 1.31 in the presence of PK2; *P* >0.1, Bonferroni’s post hoc test). Second, the EC_50_ values of both curves had no statistical difference (pH 5.96 ± 0.08 with PK2 pretreatment versus pH 5.92 ± 0.06 without PK2 pretreatment; *P* >0.1, Bonferroni’s post hoc test). Third, the threshold pH value was essentially the same.

### Intracellular mechanisms underlying the potentiation of proton-gated currents by prokineticin 2 signaling

PK2 receptors belong to the Gq-protein-coupled receptor family, and the activation of these receptors leads to a cascade of events that activate the protein kinase C (PKC) system [21,26]. To further explore whether the potentiation of I_pH_ by PK2 is mediated through the PKC signaling pathway, a selective PKC inhibitor, GF109203 X, was applied internally to DRG neurons through recording patch pipettes. In the control experiments, each neuron was patch-clamped twice with the normal internal solution. The increase of I_pH5.5_ induced by PK2 in the first patch experiment was 85.6 ± 10.7% and in the second was 81.2 ± 12.4% (n = 7, *P* >0.1, paired *t*-test). In contrast, the potentiation of I_pH5.5_ induced by PK2 was abolished when examined at 30 min after intracellular dialysis with 2 μM GF109203 X (19.9 ± 5.9%, n = 8, *P* <0.01, post hoc Bonferroni’s test; Figure 5). Phorbol 12-myristate 13-acetate (PMA, 10 μM), a membrane-permeable activator of PKC, also produced a 102.9 ± 13.2% enhanced effect on I_pH5.5_ when it was pre-applied externally to DRG neurons for 60 s. The result indicated that PKC activators can mimic the effect of PK2. Moreover, PK2 (1 nM) only caused a 7.2 ± 8.2% enhanced effect on I_pH5.5_ after FSC-231 (50 μM), a protein interacting with C-kinase 1 (PICK1) inhibitor, was applied internally to DRG neurons (n = 7, *P* <0.01, post hoc Bonferroni’s test; Figure 5). Re-patch and intracellular dialysis did not significantly alter the amplitude of I_pH5.5_. These results indicated that the potentiation of I_pH_ induced by PK2 was blocked by the PKC inhibitor and PICK1 inhibitor.

### Effect of prokineticin 2 on proton-induced membrane excitability of rat dorsal root ganglia neurons

Further experiments were performed in the presence of capsazepine (10 μM) and TTX (1 μM) to block TRPV1 and Na^+^ channel-mediated action potentials, respectively. It was shown that TTX is not effective in blocking the proton-gated currents, although it blocks the majority of voltage-gated Na^+^ currents [27]. A pH 6.0 acid stimulus induced an inward current in a DRG neuron tested with voltage-clamp recording, whereas it also produced a depolarization of the resting membrane potential in the same cell under current-clamp recording (I = 0 pA) conditions (Figure 6A). In this particular cell, pre-application of 1 nM PK2 for 60 s increased this acid-evoked depolarization of the membrane potential (Figure 6A). After exposure to pH 6.0, membrane potential depolarized from 65.1 ± 4.3 mV to 56.5 ± 4.0 mV. In contrast, PK2 increased the magnitude of acid-induced depolarization from 8.6 ± 0.7 mV to 13.4 ± 1.4 mV (paired *t*-test, *P* <0.05, n = 6). After a 20 min washout of PK2, the acid-evoked depolarization of membrane potential recovered to control condition (9.3 ± 1.4 mV, paired *t*-test, *P* >0.1, compared with 8.6 ± 0.7 mV of control condition, n = 6; Figure 6A and C).

In the presence of the TRPV1 inhibitor, capsazepine (10 μM), a steep pH drop from 7.4 to 5.5 could trigger bursts of action potentials under current-clamp conditions in a neuron tested, while the whole-cell current was also induced by pH 5.5 in the same cell with voltage-clamp recording (Figure 6B). The mean number of spikes was 1.5 ± 0.7 during exposure to pH 5.5 for 5 s in the six neurons tested (Figure 6D). Similarly, the pre-application of PK2 (1 nM, 60 s) increased the acid-induced spiking, and the mean number of spikes increased to 4.2 ± 0.8 (paired *t*-test, *P* <0.05, n = 6). After a 20 min washout with PK2, the mean number of spikes evoked by acid was 1.9 ± 0.9, which was not significantly different from the control condition (1.5 ± 0.7, paired *t*-test, *P* >0.1, n = 6; Figure 6B and D). These results indicated that PK2 increased proton-induced membrane excitability of rat DRG neurons.

### Effect of prokineticin 2 on nociceptive responses to injection of acetic acid in rats

Acetic acid elicits an intense flinch/shaking response in rats [9,24]. The flinch response mainly occurs 0 to 5 min after intraplantar injection of the acetic acid. In the present study, intraplantar injection of 20 μL acetic acid solution (0.6%) in the presence of the TRPV1 inhibitor capsazepine (100 μM) caused an intense flinch/shaking response, and treatment of 200 μM amiloride potently blocked acid-evoked pain (data not shown). Figure 7 shows that the application of PK2 dose-dependently exacerbated flinching behavior induced by acetic acid. The number of flinches increased from 11.5 ± 1.5 in control conditions to 20.3 ± 2.9 with 3 nM PK2 pretreatment (*P* <0.01, post hoc Bonferroni’s test, n = 10). The enhancing effect of PK2 on acetic acid-induced pain behavior failed to exert when PKRA (10 nM, a PK2 receptor antagonist) was co-injected together with 3 nM PK2. The number of flinches induced by acetic acid (13.1 ± 0.6) was not significantly different from that of the control conditions (*P* >0.1, post hoc Bonferroni’s test, n = 10). Moreover, intraplantar injection of PK2 or PKRA by themselves did not elicit an obvious flinch/shaking response, and the number of flinches were 0.7 ± 0.5 and 0.6 ± 0.3, respectively. These results indicate that PK2 contributes to acidosis-evoked pain.

## Discussion

The present study demonstrated that PK2 enhanced the activity of ASICs via the PK2 receptor in freshly isolated rat DRG neurons. PK2 increased the amplitude of ASIC currents, the number of spike discharges induced by acid stimuli and nociceptive responses to the injection of acetic acid. We further showed that the PKC-dependent signal pathway likely underlies intracellular mechanisms in enhancing ASIC activity by PK2.

In agreement with previous studies, extracellular application of acid solution was found to evoke three different currents in DRG neurons: slow-inactivating, rapid-inactivating, and sustained inward current [28,29]. These proton-gated currents were mediated by ASICs in the presence of the TRPV1 inhibitor capsazepine, although we did not identify the ASIC subunits in the present study. Protons activated the members of the ASIC family rather than TRPV1, since the three types of currents were blocked by amiloride, a broad-spectrum ASIC blocker. Therefore, these proton-gated currents were considered to be ASIC currents. In the present study, our results showed that PK2 enhanced all three types of proton-induced currents, and no data indicated the existence of selectivity over these acid currents recorded in DRG neurons. We showed that PK2 treatment sensitized ASIC activation by shifting the proton concentration-response curve upward and increasing the maximum response without changing the threshold value and EC_50_.

PKRs may mediate PK2 enhancing effect on proton-gated currents, since the potentiation of PK2 was blocked by PKRA, a PK2 receptor antagonist. It has been shown that PKR1 and PKR2 are present in nociceptive primary sensory neurons [15,17]. PKR1 is more abundant than PKR2 and is expressed prevalently in small neurons, while PKR2 is only expressed in a small number of medium and large neurons. It has been shown that the PK2-PKR1 pair may play a major role in pain perception [12,19,30]. However, the absence of selective antagonists for PKR1 and PKR2 means that an involvement of PKR2 cannot be ruled out in the present study. The potentiation of proton-gated currents by PK2 may involve intracellular signal transduction, since the potentiation was blocked by intracellular dialysis of GF-109203X, a selective PKC inhibitor [31]. In agreement with this, we previously found that PK2 suppresses gamma-aminobutyric-acid -activated current via the PKC signal pathway in rat primary sensory neurons [32]. Deval et al. [33] demonstrated that ASIC3-like currents in cultured DRG neurons are increased by the PKC activator, phorbol 12,13-dibutyrate, and by pain mediators such as serotonin, which are also known to activate PKC through their cognate G protein-coupled receptors.

Potentiation of ASIC currents by PKC requires the presence of PICK1, a PSD-95/Discs-large/ZO-1 homology (PDZ) domain-containing protein [34,35]. ASICs contain a PDZ-binding motif at their C termini and interact with several PDZ-containing proteins. The associated proteins control the surface expression, the subcellular distribution and the function of ASICs [36]. PICK1 has been shown to co-localize with ASICs and interact directly with the PDZ-binding domain sequences of these channels [37,38]. The PICK1 PDZ domain of ASICs may be involved in the potentiation of proton-gated currents by PK2, since the potentiation was evidently blocked by intracellular dialysis of FSC-231, a small-molecule inhibitor of the PICK1 PDZ domain [39].

ASICs are a family of cation channels and extracellular pH sensors. Activation of ASICs by protons induces an inward current, which in turn produces a depolarization of the resting membrane potential and triggers bursts of action potentials [40]. The current-clamp experiments showed that PK2 increased acid-evoked neuronal excitability of DRG neurons, which appeared to be related to its enhancing effect on the ASIC current amplitude in voltage-clamp experiments. These results are also consistent with the PK2 enhancing effect of on acetic acid-induced pain behavior in rats. In the presence of the TRPV1 inhibitor capsazepine, the pain response evoked by intraplantar injection of acetic acid solution was blocked by the ASIC blocker amiloride, suggesting the involvement of ASICs. The cutaneous acid-induced pain appears to be largely mediated by ASICs [1,41]. Thus, the enhancing effect of PK2 on acetic acid-induced pain behavior may be via ASICs rather than TRPV1. It has been shown that deletion of the PK2 and PKR1 gene strongly reduces acid-induced visceral pain [15,19].

The PK2 potentiation of ASICs activity is likely to be of physiological relevance in pathologies in which both tissue acidosis and PK2 may occur together, such as inflammation. We used the cell body of DRG neurons as a simple and accessible model to examine the characteristics of the membrane of peripheral terminals in this work. Histochemical analysis reveals the presence of ASIC2 and ASIC3 in cutaneous nerve endings [9,42]. During inflammation, protons are among the first mediators released by damaged cells, and extracellular pH values can drop to 5.4 [43]. Tissue acidosis is a common factor found in several other pain-generating conditions such as ischemia, tumor development, and skin and muscle incision following surgical procedure [44]. Protons can depolarize the peripheral free terminals of nociceptive neurons, and cause pain. In this process, ASICs play an important role although TRPVs are also likely involved. Several reports indicate that PK2 is released from inflammatory cells and levels are highly increased within inflamed tissues [18,45]. PK2 causes hyperalgesia through the activation of PKRs expressed by sensory neurons [17]. In tissue injury and inflammation, when both protons and PK2 are present, they could activate their cognate receptors and initiate nociceptive process. It is likely that PK2, as a proalgesic factor, potentiates ASIC activity.

## Conclusions

Our results indicate an important regulatory role of PK2, a new pronociceptive mediator, in nociceptive processing. PK2 increases the activity of ASICs via PK2 receptor and PKC-dependent signal pathways in rat primary sensory neurons. These findings support that PK2 signaling likely contributes to acidosis-evoked pain by sensitizing ASICs.

## Abbreviations

ANOVA, analysis of variance; ASIC, acid-sensing ion channels; DMEM, Dulbecco’s modified Eagle’s medium; DRG, dorsal root ganglion; IpH, proton-gated current; PDZ, PSD-95/Discs-large/ZO-1 homology; PICK1, protein interacting with C-kinase 1; PKC, protein kinase C; PKR, prokineticin receptor; PKRA, prokineticin receptor antagonist; PK2, prokineticin 2; TRPV1, transient receptor potential vanilloid receptor-1; TTX, tetrodotoxin.

## Competing interest

The authors declare that they have no competing interests.

## Authors’ contributions

WPH and QYZ conceived and designed the study. CYQ, FQ, YQL and JW performed and analyzed the experiments. WPH drafted the manuscript. All authors read and approved the final manuscript.
